# In Vitro and In Vivo Antitumor Activity of a Chloroform Partition from *Ibervillea sonorae* (S. Watson) Greene Endophytic *Bacillus subtilis* Extracts

**DOI:** 10.3390/plants14101474

**Published:** 2025-05-14

**Authors:** Ricardo Romero-Arguelles, César Iván Romo-Sáenz, Patricia Tamez-Guerra, Diego Fonseca-Rivera, Joel H. Elizondo-Luevano, Nancy Edith Rodriguez-Garza, Orquídea Pérez-González, Celia María Quiñones-Flores, Carlos Arzate-Quintana, Lydia Paulina Loya Hernandez, Ricardo Gomez-Flores

**Affiliations:** 1 Department of Microbiology and Immunology, School of Biological Sciences, Autonomous University of Nuevo Leon, San Nicolas de los Garza 66455, Nuevo Leon, Mexico; ricardoromeroarguelles@gmail.com (R.R.-A.); patricia.tamezgr@uanl.edu.mx (P.T.-G.); diego.fonsecarvr@uanl.com.mx (D.F.-R.); joel.elizondolv@uanl.edu.mx (J.H.E.-L.); nancy.rodriguezg@usal.es (N.E.R.-G.); operezg@uanl.edu.mx (O.P.-G.); 2 School of Medicine and Biomedical Sciences, Autonomous University of Chihuahua, Chihuahua 31109, Mexico; cquinonezf@uach.mx (C.M.Q.-F.); carzate@uach.mx (C.A.-Q.); lploya@uach.mx (L.P.L.H.); 3 Infectious and Tropical Diseases Group (e-INTRO), Salamanca Biomedical Research Institute-Tropical Diseases Research Centre of the University of Salamanca (IBSAL-CIETUS), School of Pharmacy, University of Salamanca, 37007 Salamanca, Spain

**Keywords:** *Bacillus subtilis*, endophyte, medicinal plants, lymphoma

## Abstract

Cancer is a complex disease due to its high variability and resistance to conventional treatments. The search for new therapies has prompted the study of less invasive natural sources, such as endophytic bacteria from medicinal plants. *Bacillus subtilis* is known to produce bioactive metabolites with promising pharmacological properties. This study evaluated the antitumor activity of the endophyte *B. subtilis* from *Ibervillea sonorae* against murine L5178Y-R lymphoma cells within in vitro and in vivo models. *B. subtilis* methanol extract was fractionated in hexane, chloroform, and methanol, with the chloroform partition showing the highest tumor cell growth inhibition (IC_50_ = 34.62 ± 0.180 µg/mL) and the highest selectivity index (SI = 15.53) when compared with the hexane and methanol partitions. The in vivo study showed that mice treated with 10 mg/kg of the chloroform partition significantly (*p* < 0.01) reduced the tumor volume and weight without affecting tumor-free body weight. The maximum tolerated dose test indicated that 10 mg/kg was safe and well tolerated. These results indicate that *B. subtilis* may be a promising source of selective antitumor compounds.

## 1. Introduction

Cancer continues to represent a major public health problem worldwide, with an estimated morbidity rate of 18.1 million and a mortality rate of 9.6 million per year. Some of the main characteristics of cancer include uncontrolled cell proliferation, the potential to evade control mechanisms, and cellular dimorphism [1,2]. In particular, lymphoma is a group of malignant neoplasms of lymphocytes with more than 90 subtypes, currently classified into Hodgkin’s and non-Hodgkin’s lymphoma [3]. Surgery, chemotherapy, and radiotherapy are the conventional treatments for this type of disease. However, they remain expensive and negatively impact the patient’s health due to their side effects, myelosuppression, and neurological, cardiac, pulmonary, and renal toxicity, reducing patients’ quality of life and discouraging them from undergoing current treatments, favoring the progression of cancer [4,5]. Therefore, there is a need to design new strategies that promote the control and elimination of this disease, which are more effective, selective, and less toxic than conventional therapies. The search for new drugs to treat this type of pathology has led to the discovery of new sources of bioactive molecules with selectivity and specificity characteristics [6,7].

In the last two decades, various endophytic bacteria associated with different plants have produced a wide range of bioactive compounds with therapeutic effects, especially antitumor activity [6,8]. Endophytic bacteria in cucurbits, such as *Ibervillea sonorae*, produce bioactive compounds with antitumor effects, specifically against in vitro lymphoma models [9]. This represents a source of new drugs with biological activity, especially antitumor activity. Therefore, in the present work, the antitumor potential of the chloroform partition of *B. subtilis* (ISE-B27), an endophyte of *I. sonorae*, was evaluated against murine L5178Y-R lymphoma cells (ATCC CRL-1722) in an in vitro and in vivo model using BALB/c mice.

## 2. Results

### 2.1. In Vitro Antitumor Activity of B. subtilis Extracts Against L5178Y-R Cells

**Figure 1:**
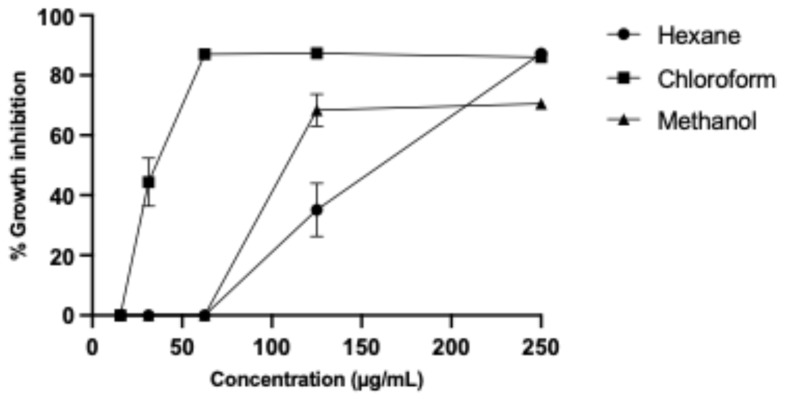
L5178Y-R lymphoma cell growth inhibition by *B. subtilis* partitions.

We obtained hexane, chloroform, and methanol partitions from *B. subtilis* (ISE-B27) crude methanol extract, resulting in the highest yield (1187 mg) for the chloroform partition, followed by the methanol partition with 1011 mg and the hexane partition with 124 mg. The partitions were evaluated against murine L5178Y-R lymphoma and human peripheral blood mononuclear cells (PBMCs), obtaining the highest IC_50_ and SI for the chloroform partition of 34.62 ± 0.180 µg/mL and 15.53, respectively (Table 1), whereas the IC_50_ of the hexane and methanol partitions were 148.1 ± 0.330 µg/mL and 117.7 ± 0.310 µg/mL; however, their respective SIs were 3.37 and 4.24 (Figure 1).

### 2.2. Biological Activity of B. subtilis Extracts

We evaluated the hemolytic and anti-hemolytic effect on erythrocytes, using the most effective and selective extract partitions against L5178Y-R cells (Table 1). The extract partition that showed the lowest hemolytic activity was chloroform with an IC_50_ of 1443 ± 0.490 µg/mL, followed by methanol-based partitioning with an IC_50_ of 223.2 ± 0.370 µg/mL, and the hexane-based partition with an IC_50_ of 74.01 ± 0.270 µg/mL. In addition, evaluation of the anti-hemolytic activity demonstrated that the methanol-based partition possessed the highest protective potential with an IC_50_ of 89.35 ± 0.290 µg/mL, whereas the hexane and chloroform partitions presented IC_50_ > 500 µg/mL (606.5 ± 0.440 and 11,847 ± 0.610 µg/mL, respectively (Table 2)). However, the partitions did not show antioxidant activity (Table 2).

### 2.3. Phytochemical Analysis of B. subtilis Extract

For the characterization of the main chemical groups present in the chloroform partition, a series of chemical tests were performed, revealing the presence and absence of various families of compounds. Table 3 shows the results of the chemical tests of the ISE-B27 (*B. subtilis*) extract chloroform partition. The test was positive for triterpenes, coumarins, tannins, and alkaloids, and negative for sesquiterpene lactones, quinones, saponins, flavonoids, and carbohydrates (Table 3).

### 2.4. Maximum Tolerated Dose of B. subtilis Extract in Tumor-Free Mice

**Figure 2:**
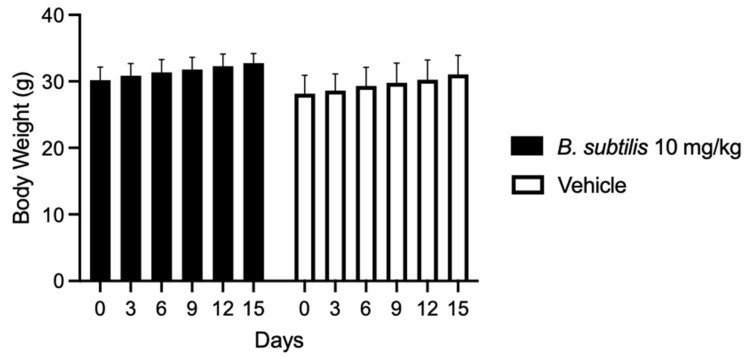
Body weight variation in mice treated with *B. subtilis* extract and vehicle for 15 days.

To determine the dose to be used in the in vivo model the maximum tolerated dose test was performed, in which the extract was administered at different concentrations. This resulted in a 100% survival of the treated group at 10 mg/kg chloroform-based partition of the ISE-B27 bacterial extract and vehicle. Furthermore, the group treated at 50 mg/kg had a survival rate of 33.3%, whereas the highest concentrations of 100 mg/kg and 1000 mg/kg were significantly toxic, resulting in mice death. Mice treated with the *B. subtilis* extract at 10 mg/kg and vehicles did not show significant differences in weight loss (Figure 2). To determine whether *B. subtilis* extract treatment does not cause liver damage, liver function was evaluated by clinical chemical analysis of the group treated with 10 mg/kg, showing no liver damage (Table 4).

### 2.5. In Vivo Effect of B. subtilis Extract Against L5178Y-R Cells

**Figure 3:**
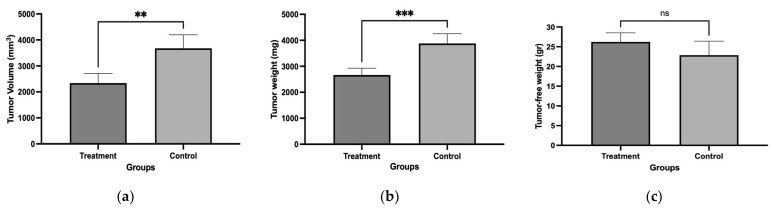
Tumor volume, tumor weight, and tumor-free weight of female BALB/c mice treated with chloroform-based partition (10 mg/kg) from ISE-B27 (*B. subtilis*) bacterial extract. (**a**) Tumor volume, (**b**) tumor weight, and (**c**) tumor-free weight. ** *p* < 0.01; *** *p* < 0.001; ns: not significant.

**Figure 4:**
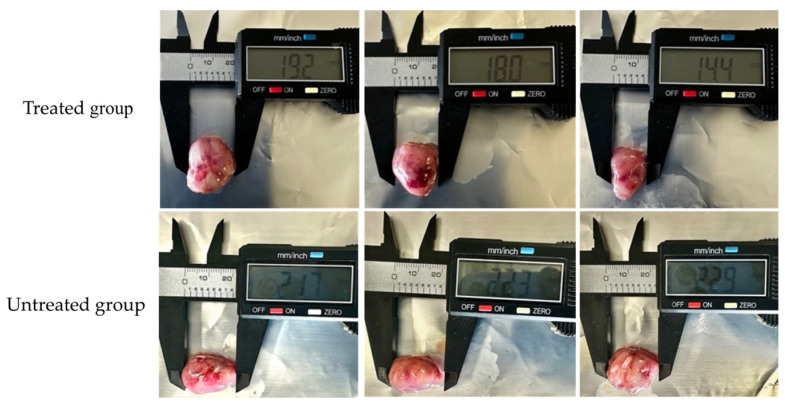
Effect of chloroform-based partition (10 mg/kg) from ISE-B27 (*B. subtilis*) bacterial extract on tumor size. Tumors of the untreated group were significantly larger compared to those of the treated group.

Mice were implanted with L5178Y-R cells and treated with the chloroform-based partition (10 mg/kg) of the bacteria ISE-B27 for 14 d after tumor implantation. We observed a significant reduction in tumor volume (*p* < 0.01) and tumor weight (*p* < 0.001) in mice treated with the chloroform partition compared to untreated tumor-bearing mice (Figure 3), without affecting tumor-free weight (Figure 4). In addition, as shown in Table 5, liver function was not affected by this treatment in tumor-bearing mice in the maximum tolerated dose test.

## 3. Discussion

Cancer represents one of the main causes of death worldwide [11]. Despite advances in cancer therapies they still have disadvantages, such as drug resistance and side effects [12], which has prompted researchers to discover new selective antitumor agents of high therapeutic efficacy and with marginal side effects [13]. Gram-positive bacteria, such as actinomycetes and endophytic *Bacillus* from medicinal plants, synthesize compounds for pharmaceutical application against cancer, such as aromatic compounds, polysaccharides, and lipopeptides [13,14,15,16].

Results of the present study demonstrated the in vitro and in vivo antitumor activity of *B. subtilis*, which is an endophyte of *I. sonorae*, a cucurbit native to northern Mexico, whose methanol and ethyl acetate extracts contain cucurbitacins, with cytotoxic properties against the tumor cell lines HepG2, HeLa, L5178Y-R, A549, M12A, LS180, and MDA-MB-231 [17,18,19,20].

Currently, the cancer incidence and mortality rate are increasing worldwide and lymphoma is one of the eleven most prevalent types of cancer [11]. Our study showed significant tumor cell growth inhibition by chloroform-based partitions of the endophytic bacterium *B. subtilis* (ISE-B27) methanol extract, exerting an IC_50_ of 34.62 ± 0.180 µg/mL against L5178Y-R cells. This result is comparable with other studies, such as one isolating a dipeptide from *Bacillus pumilus* AMK1 using chloroform which had significant cytotoxic and apoptotic activity against HepG2 liver cancer cells [21]. Another study found soluble proteins from *Bacillus thuringiensis* strains GM1, GM18, and HD-512 with cytotoxic activity against L5178Y-R cells [22].

In addition, the evaluation of hemolytic activity in the present study showed that the partition of chloroform from the bacterial extract exerted an IC_50_ of 443 ± 0.490 µg/mL, which is consistent with results obtained by others where ethyl acetate fractions did not possess hemolytic activity in human erythrocytes [23]. We also found that the chloroform partition had in vivo antitumor potential, which agrees with recent studies using a mouse xenograft model with sarcoma 180 [24]. Another study reported a reduction in tumor size and weight using a mouse xenograft of human colon cancer treated with compounds synthesized from *B. polyfermenticus* [25]. Moreover, iturin A, extracted from *B. subtilis* methanol extract, was shown to reduce 58% of the tumor weight at a dose of 3 mg/kg/day in a xenograft model of HepG2 cells [26].

Marginal information has been reported on the interaction that bacteria play in the production of bioactive compounds in medicinal plants. This study contributes to the knowledge of the relationship that these bacteria may have with the plant’s biological activity. It is then essential to investigate the compounds that exert this activity, as well as their tumor selectivity and cell death mechanisms.

## 4. Materials and Methods

### 4.1. Bacillus Strain

We used the bacterial strain *B. subtillis* ISE-B27, an endophyte of *I. sonorae* [9]. It was activated on malt extract agar (ISP2) (Sigma-Aldrich, St. Louis, MO, USA) for three days at 28 °C.

### 4.2. Preparation of Extracts

*B. subtillis* ISE-B27 was fermented in 120 mL of ISP2 broth (Sigma-Aldrich, St. Louis, MO, USA) for three days under shaking (120 rpm) at 28 ± 2 °C. Cultures were then centrifuged at 4500 rpm for 10 min, separating the biomass from the supernatant. Methanol extracts were prepared by macerating for 48 h and then suspending 50 g of the biomass in 100 mL of methanol [27]. Next, the solvent was filtered and evaporated in a rotary evaporator (Buchi R-3000; Brinkman Instruments, Inc., Westbury, Long Island, NY, USA). Fractionation was performed from the crude methanol extract in a Soxhlet apparatus with 500 mL of hexane, chloroform, and methanol for 24 h with each solvent. Once the fractionated extracts were obtained, they were evaporated and reconstituted with dimethyl sulfoxide (DMSO; Sigma-Aldrich, St. Louis, MO, USA) and stored at −20 °C until use. Extract yields were analyzed using the following formula: % yield = [(gram of dry extract/gram of biomass) (100)].

### 4.3. Cell Lines

We used the murine L5178Y-R lymphoma cell line (ATCC CRL-1722) (ATCC, Manassas, VA, USA) and human peripheral blood mononuclear cells (PBMCs) as controls, which were obtained from 20 mL of blood from a healthy volunteer donor. Blood was diluted at a ratio of 1:9 with phosphate-buffered saline (PBS) and placed in a tube containing 15 mL of Ficoll-Paque PLUS (GE Healthcare Life Sciences, Pittsburgh, PA, USA), taking care that the phases did not mix. It was then centrifuged at 400× *g* for 30 min at 20 °C, after which the lymphocyte layer was carefully collected and washed with PBS at 100× *g* for 10 min at 20 °C, the supernatant was discarded, and PBMCs were suspended in RPMI 1640 culture medium (Life Technologies, Inc., Grand Island, NY, USA) supplemented with 10% inactivated fetal bovine serum (Life Technologies, Inc., Grand Island, NY, USA) and 1% antibiotic/antimycotic solution (Life Technologies, Inc., Grand Island, NY, USA). Cells were then incubated at 37 °C in an atmosphere of 5% CO_2_ in 95% air.

### 4.4. Effect of Extracts on L5178Y-R Cell Growth

In 96-well flat-bottom plates (Corning Inc., Corning, NY, USA) in complete RPMI 1640 culture medium, L5178Y-R cell suspensions (100 μL) were cultured at a density of 1 × 10^4^ cells/well and PBMCs were cultured at a density of 1 × 10^5^ cells/well. After 24 h, cells were incubated in triplicate for 48 h at 37 °C in 5% CO_2_ with 1:2 serial dilutions of 1 mg/mL stock extracts (final concentrations ranging from 15.6 μg/mL to 500 μg/mL), in a final volume of 200 µL. Tumor cell growth was then assessed by the colorimetric 3-[4,5-dimethylthiazol-2-yl]-2,5-diphenyltetrazolium bromide reduction assay (MTT; Affymetrix, Cleveland, OH, USA), adding 15 µL/well MTT (final concentration 0.5 mg/mL). Formazan crystals were dissolved in DMSO, and optical densities (ODs) were measured at 570 nm on a MULTISKAN GO microplate reader (Thermo Fisher Scientific, Waltham, MA, USA) [28]. The percentage of cell growth inhibition was calculated as follows: % growth inhibition = 100 − ((OD_570_ in extract-treated cells/OD_570_ in untreated cells) (100)). The positive control was 0.05 µg/mL vincristine sulfate (VC; Hospira, Warwickshire, UK). Concentrations were plotted on a log scale against the percentage of growth inhibition to determine IC_50_ values, which were used to determine the selectivity index (SI). This index was calculated by dividing the IC_50_ of normal cells by that of tumor cells [29].

### 4.5. Antioxidant Activity

The antioxidant activity of each extract partition was evaluated by the 2,2-diphenyl-1-picrylhydrazyl (DPPH) method [30]. In a 96-well plate, we incubated 100 μL of extract at different concentrations and 100 μL of DPPH (Sigma-Aldrich, St. Louis, MO, USA) for 30 min at room temperature in the dark, using DMSO as a negative control and ascorbic acid as a standard at concentrations ranging from 10 µg/mL to 100 µg/mL. ODs were then read at 517 nm in a MULTISKAN GO microplate reader (Thermo Fisher Scientific, Waltham, MA, USA), and the percentage of DPPH radical inhibition was calculated.

### 4.6. Hemolytic and Anti-Hemolytic Activity

The hemolytic and anti-hemolytic activity of the extracts was determined as previously described [31]. We obtained 20 mL of blood from a healthy volunteer (approved by the Ethics Committee of the Facultad de Ciencias Biológicas, UANL protocol CI-09-2022; informed consent obtained in accordance with NOM-253-SSA1-2012) in tubes with EDTA anticoagulant, after which the erythrocytes were washed three times with PBS at pH 7.2 and 5% of erythrocytes were suspended in sterile PBS. Next, we incubated the extracts (15.625 µg/mL to 250 µg/mL) plus the erythrocyte suspension in 2 mL tubes in triplicate, using distilled water as a positive control and PBS as a negative control, at 37 °C for 30 min, after which we centrifuged at 4 °C for 5 min at 13,000 rpm. To assess the anti-hemolytic activity, we incubated a suspension of red blood cells with 150 mM of 2,2′-azobis(2-amidinopropane) dihydrochloride (AAPH) (Sigma-Aldrich, St. Louis, MO, USA) plus extracts, using PBS as a negative control and erythrocytes with AAPH as a positive control, at 37 °C for 5 h at 200 rpm and centrifuged under the conditions described above. In both cases, once the sample was centrifuged, 200 μL of the supernatant was obtained and placed in a 96-well microplate to measure the OD at 540 nm. The percentage of hemolysis and anti-hemolysis was calculated as follows: % hemolysis or % AAPH inhibition (anti-hemolysis) = [(OD_540_ treatment − OD_540_ negative control)/(OD_540_ positive control − OD_540_ negative control)] × 100.

### 4.7. Phytochemical Analysis of the Extract

The phytochemical profile of the extract was analyzed by qualitative tests to detect the presence of different compounds, using each extract dissolved in methanol. For the detection of xanthophylls, 0.2 mL of HCl was added to 0.5 mL of extract: a green or purple coloration indicated a positive result. To identify unsaturated carbon–carbon bonds we used the Baeyer test, in which 5 drops of 1% KMnO_4_ were added to 10 drops of each extract. The test is positive if a color changes from purple to reddish brown occurs together with a precipitate. Carbohydrates were detected by the Molisch test. For this, the Molisch reagent was added to 10 drops of extract and concentrated H_2_SO_4_; the presence of a purple ring at the interface was interpreted as positive. The presence of flavonoids was detected by the Shinoda test, in which 10 drops of HCl and a small piece of Mg were added to 20 drops of extracts; the presence of an orange, red, or purple color indicated a positive result. Alkaloids were detected by the Dragendorff test, in which five drops of Dragendorff reagent were added to five drops of extract: the presence of an orange-brown precipitate indicated a positive test. The phenolic hydroxyl group was determined by the FeCl_3_ test, in which two drops of reagent were added to three drops of extract; the test is positive if a red, blue, or purple color or precipitate appears. For sterols and triterpenes, the Liebermann–Burchard test was used. A total of 10 drops of the Liebermann–Burchard reagent were added to 20 drops of extract; the presence of green or blue colors was positive for sterols, whereas a purple or pink color was positive for triterpenes. Coumarins were detected by adding 10 drops of 10% NaOH to the extract, which produced a color change, followed by slowly adding 1 drop of concentrated HCl until a discoloration was observed, indicating a positive test. To determine the presence of lactones, the Baljet test was used, in which five drops of Baljet reagent were added to five drops of extract; the presence of a red, orange, or violet precipitate was a positive result [32].

### 4.8. Animals

Female BALB/c mice aged 6 to 7 wk were provided by the Bioterium of the Immunology and Virology Laboratory of the School of Biological Sciences at the Autonomous University of Nuevo Leon, Mexico. The animal study protocol was approved by the Research Ethics and Animal Welfare Committee of the Facultad de Ciencias Biológicas, Universidad Autónoma de Nuevo León (protocol code CEIBA-2021-011). They were housed in microventilated cages enriched with cardboard tubes for recreational purposes, access to water and food ad libitum, in a stress- and pathogen-free environment, and at a temperature of 22 °C, with light and dark cycles of 12 h and a relative humidity of 45%. Established guidelines for the care and welfare of animals in cancer research were followed [33]. To determine the evaluation criteria of the animals, a clinical score was used that evaluated body weight, hair condition, posture, and activity [34].

### 4.9. Maximum Tolerated Dose Test

We performed the maximum tolerated dose experiment following the protocol described above [34]. Extracts were suspended in a vehicle composed of 90% PEG-300 (Sigma-Aldrich), 5% DMSO, and 5% of 96% ethanol [35]. Three doses of 10 mg/kg of the chloroform-based extract were intraperitoneally administered to each mouse, in addition to maintaining a vehicle-treated group and the control group. The observation period was 15 d, during which the weight and clinical score of each mouse were recorded. For blood studies, we intraperitoneally administered 25 mg/kg of sodium pentobarbital (Aranda Salud Animal, Querétaro, Mexico) as an anesthetic. A cardiac puncture was then performed to obtain a blood sample, followed by euthanasia of the animal. The blood samples were stored in tubes without heparin and centrifuged at 3000 rpm for five minutes to separate the serum and perform liver function tests. Animals were euthanized by cervical dislocation if a weight loss ≥ 20% or a clinical score ≥ 3 occurred.

### 4.10. In Vivo Antitumor Activity

L5178Y-R lymphoma was maintained by intraperitoneally inoculating 0.2 mL of L5178Y-R cells (5 × 10^6^ cells/mouse) into 4- to 6-week-old female BALB/c mice as previously described [36]. Ascites cell suspensions were washed twice in PBS by centrifugation at 2000 rpm for 10 min and adjusted to 2 × 10^5^ cells/mL in PBS, after which 6- to 7-week-old female BALB/c mice received a subcutaneous administration (upper right thigh) of 0.2 mL of this lymphoma suspension. After 7 days of tumor inoculation, mice bearing the L5178Y-R tumor were selected and treated with *B. subtilis* extract. Controls were tumor-bearing mice not treated with extracts (untreated), tumor-bearing mice treated with vehicle (a solution containing 90% PEG-300, 5% DMSO, and 5% of 96% ethanol) (vehicle), and untreated normal mice (untreated control without tumor). Overall survival and tumor volume (length) (width)^2^/2 were then determined. Tumor measurements (length and width) were obtained with a vernier caliper.

### 4.11. Statistical Analysis

Results were expressed as mean ± SD of three replicate determinations per treatment (in vitro study) and five mice (in vivo study) per experimental group. Statistical analyses were performed using GraphPad Prism 9 software (GraphPad Software Inc., San Diego, CA, USA). Statistical analysis determined the distribution of the data obtained for each variable (in vivo and in vitro data) studied using the Kolmogorov–Smirnov test, and subsequently a comparison was made by analysis of variance (ANOVA). For data with a predominant normal distribution, the one-way test was used. In cases where a difference was found between the groups, the Student *t* test was used to evaluate the level of significance of the difference in the mean ± SD of the control group against each of the treatment concentrations (*p* < 0.05).

## 5. Conclusions

Results of the present study demonstrated the pharmacological potential of chloroform partitions of *Bacillus subtilis* ISE-B27 extracts, an endophyte of *Ibervillea sonorae,* and the its potential to become a promising source of compounds with therapeutic potential against cancer by it significantly reducing tumor growth without affecting the general health of mice.

## Figures and Tables

**Table 1:** In vitro performance and antitumor activity of *B. subtilis* methanol extract partitions.

Partition	Performance	L5178Y-R ^1^	PBMC ^1^	SI
Hexane	124 mg/g	148.1 ± 0.330	>500 ± 0.037	3.37
Chloroform	1187 mg/g	34.62 ± 0.180	537.7 ± 2.731	15.53
Methanol	1011 mg/g	117.7 ± 0.310	>500 ± 0.003	4.24

^1^ IC_50_ (µg/mL). Data represent the mean ± SD. Vincristine was used as a positive control, causing 80% L5178Y-R growth inhibition.

**Table 2:** Hemolytic, anti-hemolytic, and antioxidant activity of ISE-B27 extract partitions.

Partition	Hemolysis	Anti-Hemolysis (AAPH)	Antioxidant Activity (DPPH)
Hexane	74.01 ± 0.270	606.5 ± 0.440	0
Chloroform	1443 ± 0.490	11,847 ± 0.610	0
Methanol	223.2 ± 0.370	89.35 ± 0.290	0

**Table 3:** Phytochemical composition of chloroform-based partition.

Classes	Chloroform Partition
Triterpenes	++ ^1^
Coumarins	+++
Sesquiterpene lactones	-
Quinones	-
Saponins	-
Flavonoids	-
Tannins	++
Carbohydrates	-
Alkaloids	+++

^1^ ++, low intensity (50%); +++, medium intensity (75%); -, negative reaction.

**Table 4:** Liver function at 15 days following administration of treatments at the maximum tolerated dose test in tumor-free mice.

Test	Treatment (10 mg/kg)	Reference Values
Albumin	3.06 ± 0.05	2.0–4.6 g/dL [10]
Total proteins	4.06 ± 0.15	4.3–6.4 g/dL [10]
Alkaline phosphatase	174.00 ± 17.34	44–118 U/L [10]
Aspartate aminotransferase	159.5 ± 21.92	69–191 U/L [10]
Alanine transaminase	46.46 ± 4.05	26–120 U/L [10]
Total bilirubin	0.08 ± 0.02	0.3–0.8 mg/dL [10]

**Table 5:** In vivo liver function.

Test	Treated Group (10 mg/kg)	Untreated Group	Vehicle	Negative Control	Reference
Albumin	3.36 ± 0.11	3.53 ± 0.25	3.65 ± 0.05	3.10 ± 0.36	2.0–4.6 g/dL [10]
Total proteins	4.64 ± 0.15	5.26 ± 0.41	4.85 ± 0.12	4.51 ± 0.66	4.3–6.4 g/dL [10]
Alkaline phosphatase	57.40 ± 18.44	68.66 ± 30.85	127.75 ± 24.12	190.22 ± 37.12	44–118 U/L [10]
Aspartate aminotransferase	233 ± 24.33	307.66 ± 54.04	93.33 ± 12.50	140.04 ± 56.56	69–191 U/L [10]
Alanine transaminase	81.10 ± 17.34	69.29 ± 5.79	31.73 ± 2.40	67.86 ± 37.83	26–120 U/L [10]
Total bilirubin	0.08 ± 0.008	0.08 ± 0.02	0.07 ± 0.005	0.05 ± 0.01	0.3–0.8 mg/dL [10]

## Data Availability

Data are contained within the article.
